# PA-MSHA in combination with EGFR tyrosine kinase inhibitor: A new strategy to overcome the drug resistance of non-small cell lung cancer cells

**DOI:** 10.18632/oncotarget.9891

**Published:** 2016-06-07

**Authors:** Xin-min Zhao, Shi-yun Pan, Qi-ling Huang, You-ni Lu, Xiang-hua Wu, Jian-Hua Chang, Zhe-Bin Liu, Xu-Wei Cai, Qi Liu, Jia-lei Wang, Xiao-Long Fu

**Affiliations:** ^1^ Department of Medical Oncology, Fudan University Shanghai Cancer Center, Department of Oncology, Shanghai Medical College, Fudan University, Shanghai, China; ^2^ Radiation Oncology, Fudan University Shanghai Cancer Center, Department of Oncology, Shanghai Medical College, Fudan University, Shanghai, China; ^3^ Breast Surgery, Fudan University Shanghai Cancer Center, Department of Oncology, Shanghai Medical College, Fudan University, Shanghai, China; ^4^ Department of Medicine, Beijing Wanter Biopharmaceutical Co., Ltd, Huairou Yanqi Economic-Technical Development Area, Beijing, China; ^5^ Department of Radiation Oncology, Shanghai Chest Hospital, Shanghai Jiao Tong University, Shanghai, China

**Keywords:** NSCLC, PA-MSHA, apoptosis, cell cycle, EGFR

## Abstract

The inhibition of epidermal growth factor receptor (EGFR) signaling by Gefitinib provides a promising treatment strategy for non-small cell lung cancer (NSCLC); however, drug resistance to Gefitinib and other tyrosine kinase inhibitors presents a major issue. Using NSCLC cell lines with differential EGFR status, we examined the potency of PA-MSHA (Pseudomonas aeruginosa-mannose-sensitive hemagglutinin) in combination with Gefitinib on proliferation, apoptosis, cell cycle arrest, EGFR signaling and tumor growth. PC-9, A549, and NCI-H1975 cells were treated with PA-MSHA, Gefetinib, or PA-MSHA plus Gefetinib at different concentrations and times. The effects of the drugs on proliferation, cell cycle distribution and apoptosis were evaluated. The activation of EGFR and apoptotic signaling-related molecules was evaluated by Western blotting in the presence or absence of EGFR siRNA. Tumor growth and pathway signaling activation was assessed by xenografts in nude mice. A time-dependent and concentration-dependent cytotoxic effect of PA-MSHA was observed in all NSCLC cells tested. The combination of PA-MSHA plus Gefitinib enhanced the growth inhibition, sub-G1 content and apoptosis over that observed with either agent alone. Furthermore, the combination of PA-MSHA plus Gefitinib resulted in caspase-3/caspase-9 cleavage and increased inhibition of EGFR-dependent activation of AKT and ERK phosphorylation. Combination treatment was more effective in reducing tumor size and EGFR activation than either agent alone. These data suggest that PA-MSHA and Gefitinib function additively to suppress the proliferative effects of NSCLC cells of differential EGFR status. The combination of PA-MSHA and Gefitinib provides a potential new strategy to conquer drug resistance for anti-EGFR-targeted therapy of NSCLC.

## INTRODUCTION

Lung cancer was one of the most common cancers for men and women in 2012 and the most common cause of cancer-related death. It has been predicted to account for over 25% of cancer-related deaths, although there has been a slight decrease in mortality and incidence [1]. Approximately 85% of lung cancer patients have non-small-cell lung cancer (NSCLC). For early stage NSCLC, adjuvant chemotherapy is effective at improving patient disease-free survival (DFS) and overall survival (OS). For advanced-stage NSCLC, however, the response rate is only approximately 30% and the median OS of metastatic NSCLC is only approximately 12 months, despite the use of chemotherapy as first-line treatment [2].

The epidermal growth factor receptor (EGFR) is a proto-oncogene regulating cell proliferation, metastasis, and angiogenesis [3]. Abnormalities in EGFR are known to induce a strong oncogenic potential in NSCLC [4]. Tyrosine kinase inhibitors specific to EGFR (EGFR TKIs) are used in second-line and even first-line therapy in patients with metastatic NSCLC. Gefitinib and erlotinib are first-generation EGFR tyrosine kinase inhibitors (TKIs) that block the EGFR signaling pathway through reversible binding to EGFR [5]. However, the limitation of this treatment is the EGFR gene-mutation status [6]. In patients with EGFR mutations, such as the exon 19 in-frame deletion or exon 21 L858R point mutation, the initial response to first-generation EGFR TKIs is approximately 80%. Unfortunately, almost all patients acquire resistance to these agents. For 50% of these patients, resistance is derived by the occurrence of a secondary T790M mutation in exon 20 of the EGFR [7]. Recently, second-generation EGFR TKIs that inhibit EGFR activity by irreversibly binding to EGFR have been clinically developed and have shown promising anti-tumor activity in NSCLC [8]. However, these irreversible EGFR TKIs are over 100-fold less potent in NSCLC cells with the EGFR T790M mutation than in NSCLC cells with the EGFR exon 19 in-frame deletion mutation [9, 10]. A recent clinical study emphasized the limited effects of these second-generation agents, which suggests the necessity for developing a new strategy for overcoming EGFR TKIs resistance in NSCLC [11].

The interaction of humans with bacteria is among the most interesting investigative fields. It has been clearly established over the last few decades that chronic bacterial infections may contribute to carcinogenesis. Conversely, a less-obvious application of bacteria and bacteria-derived products is their use in protecting human beings from various malignant diseases. It is well known that bacteria mediate antitumor activities not only by indirect immune activation but also by direct tumoricidal effects [12–14]. For instance, a genetically modified strain of *Salmonella typhimurium* A1-R, which is auxotrophic for leu-arg and has high anti-tumor virulence, can infect tumor cells and directly cause nuclear destruction. This bacterium has been successfully used to eradicate metastases in orthotopic models of prostate, breast, and pancreatic cancer, both after local and systemic administration [15–18]. Another important example of bacterial anti-tumor action is *S. pyogenes*, which binds directly to target cells via fibronectin or collagen and eventually leads to the induction of the apoptotic process in tumor cells with complete regression of established pancreatic tumors after a single application of live *S. pyogenes* [19]. Although the antitumor effect is accompanied by massive leukocyte infiltration and elevation of pro-inflammatory cytokines, *S. pyogenes* also shows direct lytic activity against tumor cells.

*Pseudomonas Aeruginosa* injection is a type of therapeutic biological product approved in China for adjuvant treatment of patients with malignant tumors. This product is made from an inactivated mutant strain of *Pseudomonas aeruginosa* (PA-MSHA) that is characterized by rich mannose-sensitive hemagglutination pili (type 1 fimbriae). PA-MSHA has been successfully used in clinical cancer therapy for many years, although its detailed mechanism of action remains unclear. In recent studies, PA-MSHA has been shown to directly inhibit tumor cell proliferation in vitro and induce apoptosis in human hepatocarcinoma, nasopharyngeal cancer and breast cancer cells [20, 21]. Interestingly, an in-depth study demonstrated that the mannose-mediated EGFR signaling pathway is involved in the apoptosis of breast cancer cells (MDA-MB-231HM and MDA-MB-468) induced by PA-MSHA [22]. These results imply the potential therapeutic value of PA-MSHA in tumors typically associated with EGFR over-expression and mutations.

In this study, to examine the effects of PA-MSHA we selected three different NSCLC cell lines based on their different gene-expression status: A549 is an EGFR wild type cell line with primary EGFR-TKI resistance, PC-9 is an EGFR-TKI-sensitive cell line with an exon 19 deletion mutation, and NCI-H1975 is an acquired EGFR-TKI-resistant cell line with T790M and L858R mutations. To evaluate the potential of PA-MSHA to assist in overcoming EGFR-TKI drug resistance, we observed the cell growth inhibition, apoptosis induction, and cell cycle redistribution of these three cell lines after administration of PA-MSHA alone or in combination with Gefitinib. Our results suggest that the use of a combination PA-MSHA and Gefitanib represents a possible tool in an adjuvant or metastatic setting for NSCLC.

## RESULTS

### Effect of PA-MSHA in combination with Gefitinib on the proliferation of NSCLC cell lines

**Figure 1:**
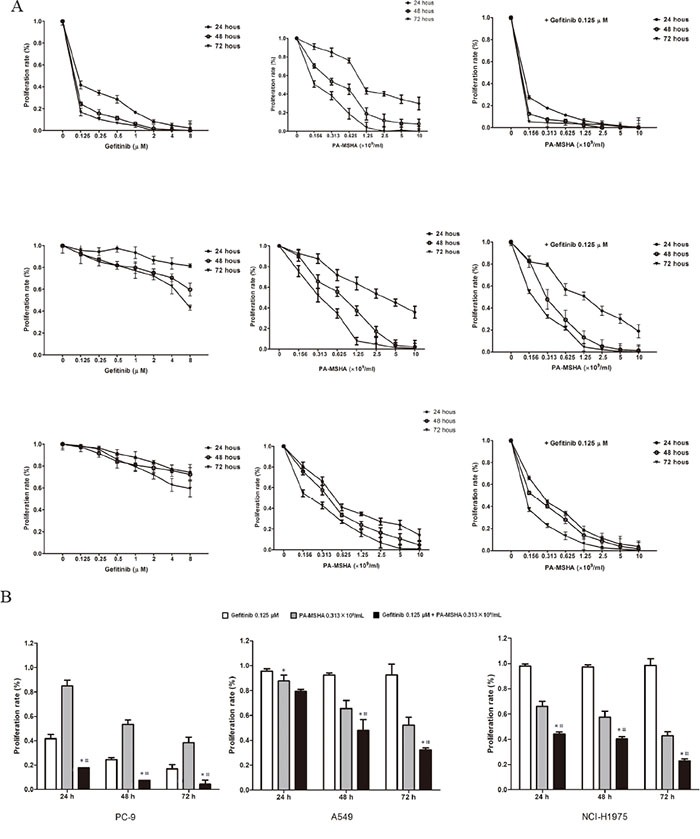
Effect of Gefitinib, PA-MSHA or a combination of the two drugs on the proliferation of NSCLC cells **A.** A549, PC-9, and NCI-H1975 cells were treated with increasing doses of Gefitinib, PA-MSHA, or Gefitinib plus PA-MSHA at the indicated concentrations for 24, 48, and 72 hours. Cell proliferation was measured by MTT assay and expressed as the proliferation rate. **B.** The proliferation of cells treated with one dose of Gefitinib and/or PA-MSHA is shown as a histogram. The data are presented as means ± SD of triplicates from one representative experiment and are representative of 3 independent experiments. *, Gefitinib + PA-MSHA *vs.* Gefitinib; #, Gefitinib + PA-MSHA *vs.* PA-MSHA, *P*<0.05.

To investigate the effect of PA-MSHA alone and in combination with Gefitinib, we examined three human NSCLC cell lines with varying genetic EGFR status and differential corresponding sensitivity to EGFR-TKIs: PC-9 (sensitive), A549 (primary resistant), and NCI-H1975 (acquired resistant). As expected, proliferation was inhibited with increasing doses of Gefitinib, but the inhibition rate was higher for PC-9 cells than for A549 or NCI-H1975 cells. However, PA-MSHA produced substantial dose- and time-dependent growth inhibition in all three cell lines, regardless of their sensitivity to Gefitinib. Combining various concentrations of PA-MSHA with 0.125 μM Gefitinib resulted in more pronounced growth inhibition than Gefitinib alone, particularly for A549 and NCI-H1975 cells (Figure 1A). To determine whether the effect is synergistic, 0.125 μM of Gefitinib plus 0.313×10^9^/ml of PA-MSHA were compared with Gefitinib or PA-MSHA alone. As shown in Figure 1B, for all three NSCLC cell lines, the proliferation rates for PA-MSHA combined with Gefitinib were significantly lower than those for Gefitinib or PA-MSHA alone (*P*<0.05), with the possible exception of A549 cells at 24 hours. These results indicate that PA-MSHA enhances the growth inhibition by Gefitinib of NSCLC cells, especially for EGFR-TKI-resistant A549 and NCI-H1975 cells.

### Effect of PA-MSHA in combination with Gefitinib on the redistribution of the cell cycle in NSCLC cell lines

**Figure 2:**
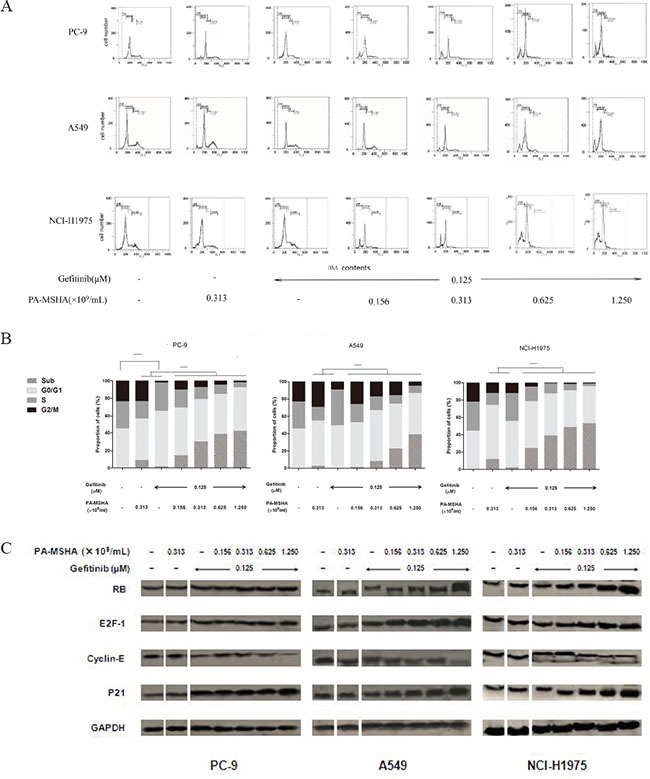
Effect of Gefitinib, PA-MSHA or a combination of the two drugs on the cell cycle distribution and proteins associated with cell cycle control in NSCLC cells A549, PC-9, and NCI-H1975 cells were treated with Gefitinib, PA-MSHA, or Gefitinib plus PA-MSHA at the indicated doses for 24 hours. **A.** The cell cycle distribution (sub-G1, G0/G1, S, and G2/M) was determined by flow cytometry; and **B.** the proportions of cells in each phase were expressed as percentages. The experiments were performed three times with similar results. *, *P*<0.05. **C.** Levels of RB, E2F-1, Cyclin-E, and P21 were determined by Western blotting. GAPDH was tested as a loading control. Results are representative of three independent experiments. The split panels on the blots are from the same membrane with the same exposure.

To determine whether the effects of Gefitinib and PA-MSHA on cell proliferation might be explained in part by effects on the cell cycle, cells were treated with PA-MSHA, Gefitinib or Gefitinib plus increasing doses of PA-MSHA for 12 hours and then were stained with PI and analyzed by flow cytometry (Figure 2A and 2B). PA-MSHA alone arrested cells in the G1 phase (Sub and G0/G1) and decreased the proportion of cells in the S phase. Furthermore, Gefitinib alone promoted a significant increase in the proportion of PC-9 cells in the G0/G1 phase and a reduction in the proportion in the G2/M phase, though the effects were less obvious for A-549 and NCI-H1975 cells. However, Gefitinib in combination with PA-MSHA resulted in a more potent cell cycle redistribution effect, especially for the EGFR-TKI-resistant A-549 and NCI-H1975 cells. In all three NSCLC cell lines, combination treatment at a range of doses of PA-MSHA as compared with Gefitinib alone (*P*<0.05) led to an increased proportion of cells in the Sub-G1 phase and a decreased proportion of cells in the S or G2/M phases.

We further evaluated the effects of this drug combination by examining levels of RB, E2F-1, P21, and Cyclin E, each of which have established roles in cell cycle regulation. PA-MSHA alone had minimal effects on the levels of these proteins in PC-9, A549, and NCI-H1975 cells. Furthermore, the effects of Gefitinib alone were only observed for PC-9 cells, which showed a modest but reproducible increase in RB, E2F-1, and P21, with a decrease in Cyclin E. However, regardless of EGFR-TKI sensitivity, Gefitinib combined with PA-MSHA significantly increased RB, E2F-1, and P21 expression and decreased Cyclin E expression (Figure 2C). These results are consistent with an effect of PA-MSHA in enhancing cell cycle arrest by Gefitinib.

### Effect of PA-MSHA in combination with Gefitinib on apoptosis

**Figure 3:**
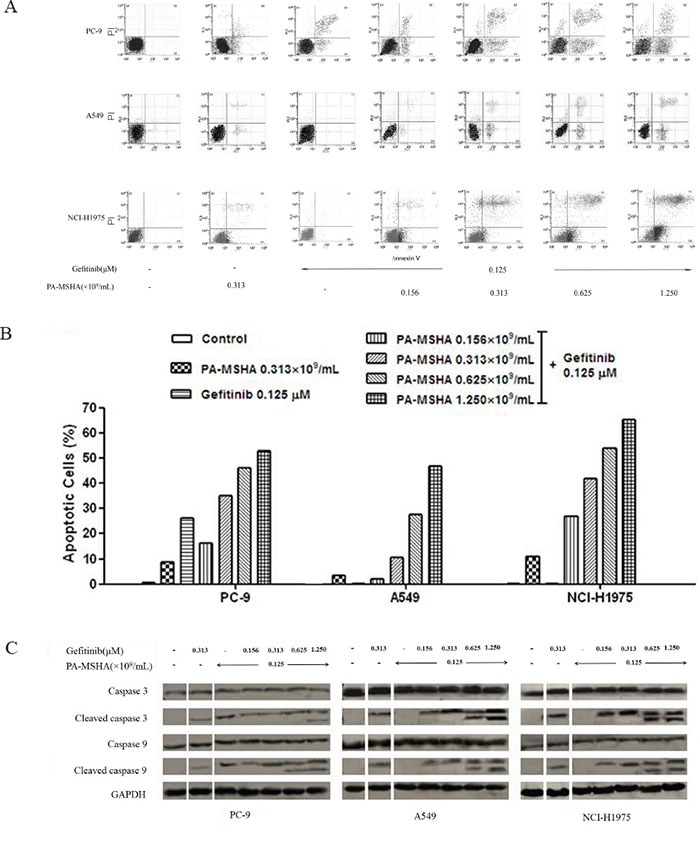
Effect of Gefitinib, PA-MSHA or a combination of the two drugs on the induction of NSCLC cell apoptosis and caspase expression A549, PC-9, and NCI-H1975 cells were treated with Gefitinib, PA-MSHA, or Gefitinib plus PA-MSHA at the indicated doses for 12 hours. **A.** The apoptotic cells were detected by annexin V/propidium iodide staining; and **B.** the ratio of apoptotic cells in each group were expressed as percentages. The experiments were performed three times with similar results. **C.** Levels of caspase-3, caspase-9, and their cleaved forms were determined by Western blotting after 24 hours drug treatment. GAPDH was tested as a loading control. Results are representative of three independent experiments. The split panels on the blots are from the same membrane with the same exposure.

In our previous study, PA-MSHA was found to induce early and late apoptosis in A-549, PC-9, and NCI-H1975 cells. To determine whether PA-MSHA can enhance the effects of Gefitinib on NSCLC cell apoptosis, cells were treated for 12 hours with PA-MSHA, Gefitinib, or Gefitinib in combination with increasing doses of PA-MSHA and then were stained with annexin V/PI and analyzed by flow cytometry (Figure 3A and 3B). Whereas apoptosis was increased to some extent for all three cell lines after treatment with PA-MSHA, Gefitinib only promoted an obvious level of apoptosis (26.7%) in PC-9 cells. Furthermore, for all three NSCLC cell lines, the percentages of apoptosis in the PA-MSHA plus Gefitinib group were significantly increased in a PA-MSHA concentration-dependent manner, suggesting that the effect of the drug combination occurs regardless of the EGFR-TKI resistance status.

The caspase family proteins were examined to investigate the underlying mechanism of apoptosis induced by Gefitinib, PA-MSHA, and their combination. The lysates were analyzed using antibodies against caspase-3, caspase-9, and their active cleaved forms. With PA-MSHA alone, the expression of cleaved caspase-3 and −9 proteins was observed in all three cell lines, but with Gefitinib alone, cleaved caspase-3 and −9 proteins were only observed in PC-9 cells, potentially due to the EGFR-TKI resistance of A549 and NCI-H1975 cells. However, in combination with Gefitinib, PA-MSHA induced a dose-dependent increase in cleaved caspase-3 and −9 in A549 and NCI-H1975 cells (Figure 3C). These results verify that treatment with PA-MSHA can reverse the EGFR-TKI resistance of NSCLC cell lines.

### Effect of PA-MSHA in combination with Gefitinib on EGFR signaling

**Figure 4:**
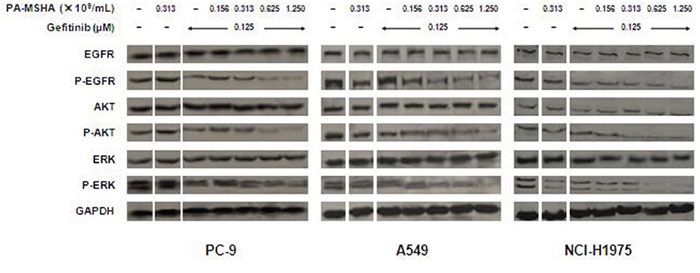
Effect of Gefitinib, PA-MSHA or the two drugs in combination on the EGFR signaling pathway in NSCLC cells A549, PC-9, and NCI-H1975 cells were treated with Gefitinib, PA-MSHA, or Gefitinib plus PA-MSHA at the indicated doses for 24 hours. Levels of EGFR, AKT, ERK, and their phosphorylated forms were determined by Western blotting, with GAPDH as a loading control. Results are representative of three independent experiments. The split panels on the blots are from the same membrane with the same exposure.

To characterize whether the growth inhibition induced by Gefitinib and PA-MSHA might involve effects on EGFR signaling, we examined the expression of several key regulators that function within the EGFR signaling pathway. As shown in Figure 4, PA-MSHA had little or no effect on the expression of p-EGFR, p-AKT, and p-ERK. Gefitinib alone promoted a reduction in the expression of these phosphorylated proteins in PC-9, but not A549 and NCI-H1957 cells. Moreover, the combination of Gefitinib and increasing doses of PA-MSHA further reduced the expression of phosphorylated proteins with minimal effect on the total protein levels in PC-9, as well as EGFR-TKI-resistant A549 and NCI-H1975 cells. These results suggest that EGFR signaling may play an important role in the combined effects of PA-MSHA with Gefitinib, especially for EGFR-TKI-resistant NSCLC.

**Figure 5:**
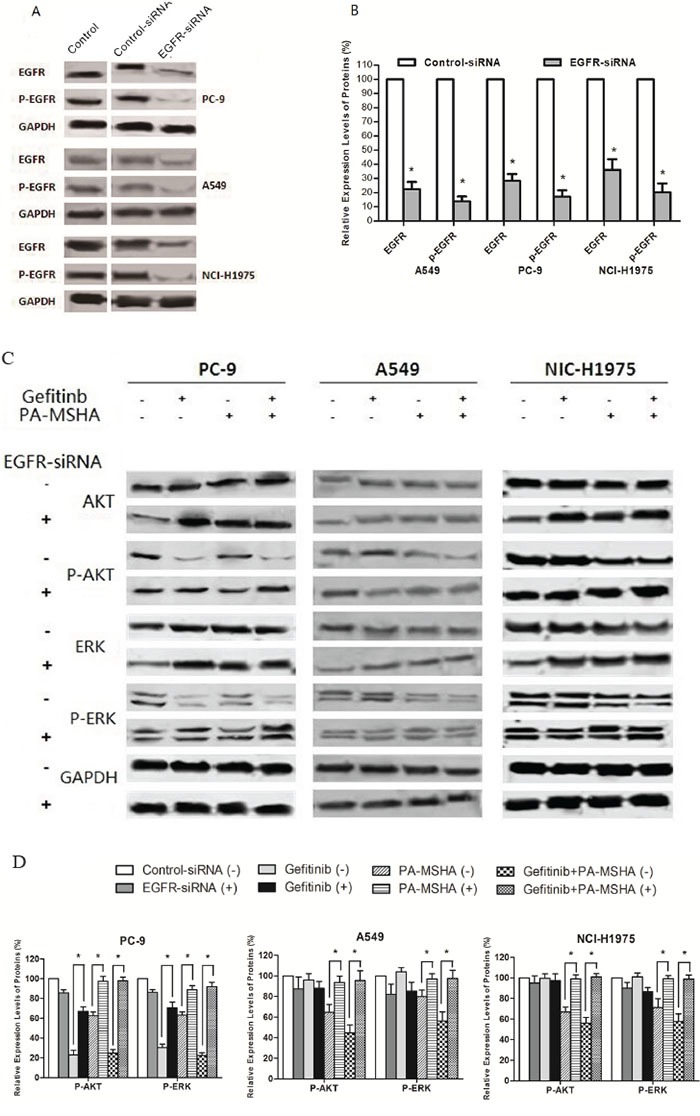
Effect of reduced EGFR expression on signaling pathway regulation by Gefitinib and/or PA-MSHA **A.** Representative experiment showing EGFR levels in untransfected cells (Control) or 72 hours after transfection with control siRNA or EGFR-siRNA. **B.** Quantification of triplicate experiments from panel A. **C.** Effects of silencing EGFR on the levels of AKT, ERK, and their phosphorylated forms were determined by Western blotting after 72 hours transfection with control siRNA (−) or EGFR siRNA(+); and 48 hours treatment with 0.125 μM Gefitinib and/or 0.313×10^9^/ml of PA-MSHA. **D.** The ratios of phosphorylated/total protein were quantified by densitometry of triplicate experiments. GAPDH was tested as a loading control. *, *P*<0.05 *vs* control-siRNA-transfected cells.

To verify the role of EGFR signaling in PA-MSHA/Gefitinib treatment, we used siRNA to reduce EGFR levels by RNA interference. As shown in Figure 5A and 5B, the siRNA was ~80% effective in reducing EGFR protein levels at 72 hours post-transfection in the three cell lines (*P*<0.05). A similar reduction in EGFR and p-EGFR levels was observed after an additional 48 hours exposure to 0.313×10^9^ cells/ml of PA-MSHA, 0.125μM Gefitinib or Gefitinib plus PA-MSHA (data not shown). Transfection with EGFR siRNA compared with control siRNA significantly weakened the effect of Gefitinib plus PA-MSHA in reducing pAKT and pERK levels in the three cell lines (Figure 5C and 5D). These findings suggest that the effects of Gefitinib and PA-MSHA on p-AKT and p-ERK are mediated by reduced EGFR signaling.

### Effect of PA-MSHA in combination with Gefitinib on tumor growth

**Figure 6:**
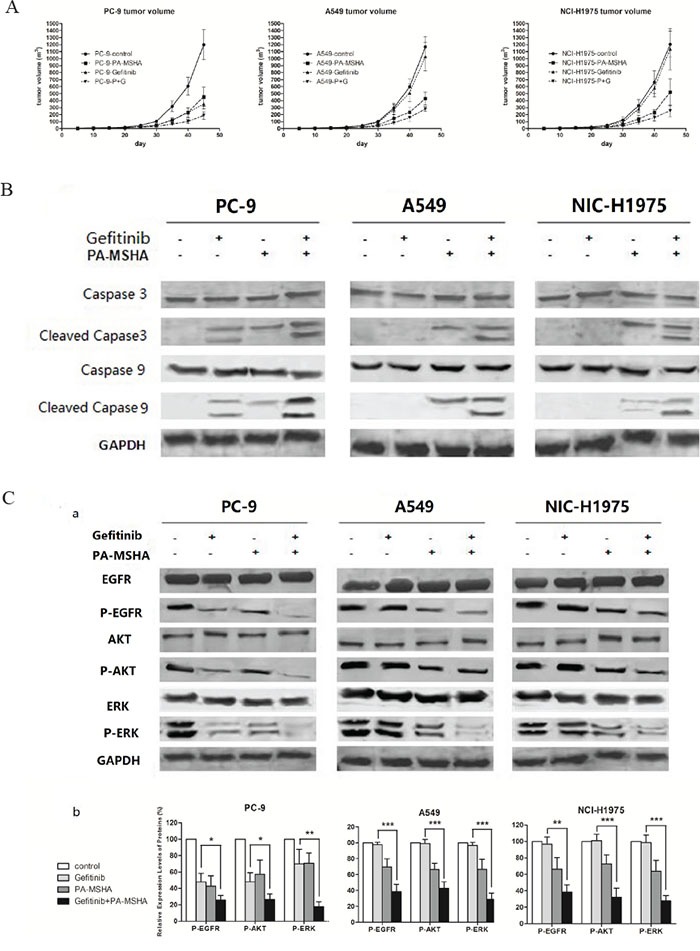
The effect of the administration of Gefitinib, PA-MSHA, or a combination of the two drugs on tumor growth of PC-9-, A549-, or NCI-H1975-xenotransplanted nude mice **A.** Mean tumor volume was measured at the indicated number of days after transplantation. **B.** The expression levels of caspase-3, caspase-9, and their cleaved forms were determined by Western blotting, with GAPDH as a loading control. **C.** The expression levels of EGFR, AKT, ERK, and their phosphorylated forms were determined by Western blotting (a); and the ratios of phosphorylated/total protein were quantified by densitometry of triplicate experiments (b), *, *P*<0.05; **, *P*<0.01; ***, *P*<0.001.

To determine whether the combination of Gefitinib plus PA-MSHA is effective in reducing NSCLC tumor growth in vivo, we assessed tumor growth after transplantation of PC-9, A549, and NIC-H1975 cells into nude mice. Consistent with the in vitro results, the administration of Gefitinib reduced the growth only for PC-9 cells, while PA-MSHA reduced the growth to some extent for all three NSCLC cell lines. Furthermore, Gefitinib plus PA-MSHA was the most effective in reducing the tumor volume for all three cell lines, with 80–70% reduction (Figure 6A).

To assess the in vivo effects of the drug combination on apoptotic and EGFR signaling pathway proteins, we preformed Western blotting of lysates from excised tumor xenografts after treatment. Consistent with the in vitro results, an increase in cleaved caspase-3 and −9 were observed for all three types of xenografts by treatment with Gefitinib plus PA-MSHA (Figure 6B). Additionally, there was a trend toward a significantly lower level of pEGFR, pAKT, and pERK in tumor issues from the combination group (Figure 6C). These results confirm the in vitro results suggesting that PA-MSHA enhances the antiproliferative and apoptotic effects of Gefitinib through the suppression of EGFR signaling.

## DISCUSSION

To date, no approved therapies exist for metastatic NSCLC patients who fail first generation reversible EGFR-TKIs. The most common mechanisms of resistance to Gefitinib and Erlotinib therapies are wild type EGFR status (primary resistance) or development of a secondary gatekeeper mutation, T790M (acquired resistance). Although Afatinib was expected to overcome EGFRT790M-mediated acquired resistance to first-generation reversible EGFR TKIs, a recent phase III study of Afatinib failed to demonstrate overall survival improvement in Gefitinib or Erlotinib-resistant patients, with a response rate of less than 10% [10]. Therefore, the treatment options for NSCLC remain unsatisfactory.

In this study, we sought to determine whether PA-MSHA might reverse the drug resistance of EGFR-TKIs in NSCLC. We evaluated the anti-cancer effects in vitro and in vivo to three different genotypes of NSCLC cell lines by Gefitinib in combination with PA-MSHA. Our data show that Gefitinib plus PA-MSHA can inhibit cell proliferation, redistribute the cell cycle, induce apoptosis and inhibit tumor growth in PC-9, A549, and NCI-H1975 cells. These results are supported by the reduced tumor growth in xenotransplanted nude mice after combination treatment. This phenomenon suggests that PA-MSHA can recover the sensitivity of Gefitinib in EGFR-TKI-resistant cells.

Previous in vitro studies reported that PA-MSHA alone can directly inhibit proliferation of breast cancer cells and gastric cancer cells [21–23]. We have extended these findings to NSCLC, and have also demonstrated that Gefitinib plus PA-MSHA is more effective than Gefitinib or PA-MSHA alone at decreasing the proliferation of PC-9, A549, and NCI-H1975 cells. On the basis of this phenomenon, we deduce that PA-MSHA may have synergistic effects with Gefitinib. Our data suggest that the growth suppression and cell death induced by PA-MSHA may occur despite the EGFR gene mutation status.

Consistent with previous results [20–22], a decrease in the S phase population and an increase in the sub-G1 population were observed in PA-MSHA-treated cells. When using Gefitinib plus PA-MSHA, these effects were enhanced to some extent. Western blots demonstrated that RB, P21, and E2F-1 increased more apparently in all three cell lines after treatment with Gefitinib combined with PA-MSHA, which provides an explanation of why the cells arrested in the sub-G1 phase [24]. Conversely, Cyclin E, which plays a major positive role in the G0/G1 switch to S phase [25, 26], decreased more apparently. Therefore, the efficacy of the combination of Gefitinib and PA-MSHA may be derived in part from effects on molecular regulators that mediate G0/G1 arrest.

Earlier data demonstrated that apoptosis activated via caspases plays a critical role in the carcinogenesis, pathogenesis, etiology, and therapy of several human malignancies [20–22, 27]. Apoptosis has been shown to be initiated and executed through two main pathways, the intrinsic and extrinsic pathway [28, 29]. In the intrinsic pathway, also called mitochondrial pathway, downstream cleavage of caspase-9 and caspase-3 is activated [29, 30]. Our data demonstrate that apoptosis of NSCLC cells induced by PA-MSHA alone or in combination with Gefitinib is mediated directly via caspase-3 and −9, suggesting that the intrinsic pathway mediated by mitochondria may play an important role in the apoptosis triggered by the two drugs in combination. These results were confirmed using an in vivo xenograft model, further supporting the potential role of apoptosis following treatment with the Gefitinib and PA-MSHA in combination.

The EGFR signaling pathway plays a critical role in proliferation, invasion, and survival in the development and progression of NSCLC [3, 4]. PI3K/Akt/mTOR and RAS/RAF/MEK/ERK are two main downstream pathways of EGFR signaling. In this study, EGFR, Akt, ERK, and their phosphorylated forms were selected for examination as potential mediators of Gefitinib and/or PA-MSHA signaling through the EGFR in NSCLC. In PC-9 and NCI-H1975 cells, Gefitinib alone can inhibit the EGFR signal pathway; however, when PA-MSHA was used in combination with Gefitinib, EGFR inhibition was observed in all three NSCLC cell lines, both in vitro and in vivo. Our data suggest that PA-MSHA may provide a new gateway toward overcoming EGFR-TKI resistance in NSCLC.

Our above results showed the gefitinib and PA-MSHA could suppress EGFR-mediated p-AKT and p-ERK pathway in three lung cancer cell lines, including A549. However, since the expression of EGFR is much lower in A549 cells than other lung cancer cells [31], it is possible that PA-MSHA inhibit the activities of AKT or ERK of A549 through other pathways rather than EGFR signaling. A recent study demonstrated that PA-MSHA could directly induce G0-G1 cell cycle arrest of the cancer cells [32], suggesting the combination of PA-MSHA and gefitinib inhibits lung cancer cell proliferation through an EGFR independent pathway. In addition, PA-MSHA has been reported to induce endoplasmic reticulum (ER) stress in breast cancer cell lines through the IRE1 signaling pathway [33] indicating that combination of PA-MSHA and tyrosine kinase inhibitors may promote cancer cells apoptosis through up-regulation of the endoplasmic reticulum (ER) stress-induced apoptosis pathways. In our next project, we will focus on detailed mechanisms through which PA-MSHA plus TKI could enforce a synergistically inhibitory effect on lung cancer cells”

PA-MSHA has been shown to directly inhibit tumor cell proliferation in vitro and induce apoptosis in human hepatocarcinoma, nasopharyngeal cancer and breast cancer cells [31]. However, the molecular mechanisms are still under investigation. Gefitinib blocks signal transduction pathways responsible for the proliferation and survival of cancer cells. In addition, evidences suggest that there might be crosstalk of signal pathways down stream of gefitinib and PA-MSHA. It has been reported that PA-MSHA induces significant cell proliferation inhibition and cell cycle arrest of hepatocarcinoma cells through decreasing the levels of cyclins D1, cyclins E, CDK2, CDK4, and increasing the level of p21 and p27 [32]. The similar inhibitory effects on cancer cell cycle by gefitinib through increased expression of p27(Kip1) cyclin-dependent kinase inhibitor and decreased expression of aurora B have been reported [34]. Moreover, PA-MSHA could inhibit Akt/IκBβ/NF-κB pathway, which are well-studied downstream signals of EGFR. Taken together, previous publication and our study suggest there is crosstalk of signal pathways down stream of gefitinib and PA-MSHA.

There are still some limitations of our study. First, we did not investigate one mechanism of acquired resistance of EGFR-TKIs-c-MET gene amplification—either alone or coexisting with other mutations, such as T790 M. Second, the crosstalk effect of the EGFR pathway and other signaling pathways need to be verified with detailed experiments. Nevertheless, these results provide the foundations for further work to specify mechanisms of cross-talk between these two pathways, including effects on potential unknown targets.

In conclusion, our data demonstrate for the first time that Gefitinib in combination PA-MSHA promotes anti-proliferation, cell cycle redistribution, apoptosis inducement via caspase family proteins, and inhibition of EGFR signal pathway effects in different genotypic NSCLC cells, and especially in EGFR-TKI-resistant (A549 and NCI-H1975) cells. In addition to the downstream effects of immune activation, cytotoxicity may contribute to the efficacy of PA-MSHA in NSCLC treatment. PA-MSHA, either alone or in combination with Gefitinib, could provide a novel strategy for the management of EGFR-TKI-resistant NSCLC.

## MATERIALS AND METHODS

### Cell lines, materials, and antibodies

Human NSCLC cell lines (PC-9, A-549, and NCI-H1975) were obtained from the American Type Culture Collection (ATCC, Manassas, VA, USA). The PC-9 cell line has a high sensitivity for EGFR-TKIs and an exon 19 deletion; A549 is a primary cell line resistant to EGFR-TKIs with wild-type EGFR; and NCI-H1975 has acquired resistance to EGFR-TKIs with T790M (exon 20) and L858R (exon 21) point mutations. All cell lines were cultured in DMEM medium (Gibco, San Francisco, CA, USA) supplemented with 10% heat-inactivated (56°C, 30 minutes) fetal calf serum (PAA, Pasching, KA, Austria), 2 mmol/L glutamine, penicillin (100 U/ml) and streptomycin (100 μg/ml) (Gibco). The cells were cultured at 37°C with 5% CO_2_ in a humidified atmosphere.

The strain of PA-MSHA used in this study was kindly provided by Wanter Biopharma Company (Beijing, China). The PA-MSHA was scale-cultured at 37°C for 24 h, inactivated by a chemical method and purified by centrifugation.

Primary rabbit or mouse antibodies against the proteins were from Cell Signaling Technology (Danvers, MA, USA): caspase-3 (#9662), caspase-9 (#9502), EGF receptor (1F4) (#2239), phospho-EGF receptor (Thr669) (D2F1) (#8808), Akt (#9272), phospho-Akt (Ser473) (#9271), p44/42 MAPK (Erk1/2) (137F5) (#4695), phospho-p44/42 MAPK (Erk1/2) (Thr202/Tyr204) (#9101), p21 Waf1/Cip1 (DCS60) (#2946), Rb (4H1) (#9309), Cyclin E1 (HE12) (#4129), E2F-1 (#3742), and GAPDH (14C10) (#2118).

### Cell proliferation

The effects of PA-MSHA on the survival of NSCLC cells were determined by 3-(4,5-dimethylthiazol-2-yl)-2,5-diphenyltetrazolium bromide (MTT) assay. PC-9, A549, and NCI-H1975 cells were seeded in 96-well plates (1×10^4^ cells/well) and then treated with PA-MSHA alone (10, 5, 2.5, 1.25, 0.625, 0.313, or 0.156×10^9^/ml), Gefitinib alone (8, 4, 2, 1, 0.5, 0.25, or 0.125 μM), or 0.125 μM Gefitinib plus PA-MSHA (10, 5, 2.5, 1.25, 0.625, 0.313, or 0.156×10^9^/ml) for different times (0, 24, 48, or 72 hours). MTT was then added to the cells for another 4 hours. After removal of the culture medium, the remaining MTT formazan crystals were dissolved with DMSO and measured at 490 nm using a microplate reader. The percentage of inhibition was calculated as follows: Inhibition ratio (IR) (%)=(1−OD_sample_/OD_control_)×100%. Experiments were carried out in triplicate, and the IC50 (the concentration of drug that inhibits cell growth by 50%) values at 24 hours were determined.

### Flow cytometry with annexin V-FITC and propidium iodide staining

Cells (10^6^/ml) were seeded in 6-well plates and reached 70–80% confluence after 6 hours in culture. Without changing the FBS-supplemented media, cells were treated for 12 hours with PA-MSHA alone (0.156, 0.313, 0.625, or 1.25×10^9^/ml), Gefitinib alone (8, 4, 2, 1, 0.5, 0.25, or 0.125 μM), or 0.125 μM Gefitinib plus PA-MSHA (10, 5, 2.5, 1.25, 0.625, 0.313, or 0.156×10^9^/ml). Subsequently, cells were subjected to an annexin V/propidium iodide (PI) dual staining assay according to the manufacturer's protocol. Stained cells were analyzed with a fluorescence activating cell sorter (Becton Dickinson, CA, USA), and the percentage of apoptotic cells was determined using ModFit LT 3.0 software (Becton Dickinson, CA, USA).

### Western blotting

Cells were directly lysed in lysis buffer containing 2 M sodium chloride, 10% NP-40, 10% SDS, 1 M Tris-Cl, 1 g/L phenyl-methylsulfonyl fluoride (PMSF), 0.1 g/L aprotinin and 0.01 g/L leupeptin. The cell lysates were subjected to SDS-PAGE electrophoresis and then blotted onto polyvinyl difluoride membranes. After the membranes were blocked with BSA for 1 hour, the expression of various proteins was detected using primary (1/1000) and secondary antibodies conjugated with horseradish peroxidase (1/800) and enhanced chemiluminescence reagents (Pharmacia, Buckinghamshire, UK).

### RNA interference

siRNA against EGFR or negative control siRNA (100 nM) was transfected into A549, PC-9 and NCI-H1975 cells according to the transfection protocol of Lipofectamine 2000 (Invitrogen, Shanghai, China). The target sequences were as follows: EGFR-siRNA, sense 5′-UGA UCU GUC ACCACA UAA UUA CGG G-3′ (EGFR-HSS103114); nonspecific control siRNA, sense 5′-GGA GCU GCC CAU GAGAAA UUU-3′. Seventy-two hours after transfection, knockdown was assessed by Western blotting of parallel transfection reactions. To determine the effects of EGFR-siRNA on the PA-MSHA-induced down regulation of total and phosphorylated levels of AKT and ERK, cells were transfected with EGFR-siRNA or control-siRNA for 72 hours and then treated with 0.313×10^9^ cells/ml of PA-MSHA, 0.125 μM Gefitinib or Gefitinib plus PA-MSHA before Western blot detection.

### In vivo tumor xenograft study

Four- to six-week-old female BALB/c-nu/nu nude mice were purchased from the Shanghai Institute of Materia Medica, Chinese Academy of Sciences (Shanghai, China). Tumors were initiated by subcutaneous (s.c.) injection of 5×10^6^ cells into the right mammary fat pad of nude mice. The mice were randomly assigned to the control or experimental groups. Mice were given daily s.c. treatments of 0.1 ml PBS (control) or 0.1 ml PA-MSHA (2.2×10^10^ cells/ml); and/or five days per week treatment of Gefitinib suspended in 1% Tween 80 at 1 mg/kg/day by oral gavage. Tumors were measured twice a week with microcalipers, and tumor volumes were calculated using the formula π/6×larger diameter×(smaller diameter)^2^. At the end of the experiment, tumors were dissected and snap-frozen. Total protein was extracted from the tumors to quantify the level of proteins by Western blotting.

### Statistical analysis

Statistical analysis was performed using the Statistical Package for the Social Sciences (SPSS) Version 13 software for Windows (SPSS Inc., Chicago, IL, USA). Data from 3 to 5 independent experiments were calculated as means and standard deviations. The significance of differences between experimental conditions was determined using the two-tailed Student's t-test. P-values of less than 0.05 were considered significant.
